# A Multi-Atlas Labeling Approach for Identifying Subject-Specific Functional Regions of Interest

**DOI:** 10.1371/journal.pone.0146868

**Published:** 2016-01-21

**Authors:** Lijie Huang, Guangfu Zhou, Zhaoguo Liu, Xiaobin Dang, Zetian Yang, Xiang-Zhen Kong, Xu Wang, Yiying Song, Zonglei Zhen, Jia Liu

**Affiliations:** 1 State Key Laboratory of Cognitive Neuroscience and Learning & IDG/McGovern Institute for Brain Research, Beijing Normal University, Beijing, 100875, China; 2 Beijing Key Laboratory of Applied Experimental Psychology, School of Psychology, Beijing Normal University, Beijing, 100875, China; Chinese Academy of Sciences, CHINA

## Abstract

The functional region of interest (fROI) approach has increasingly become a favored methodology in functional magnetic resonance imaging (fMRI) because it can circumvent inter-subject anatomical and functional variability, and thus increase the sensitivity and functional resolution of fMRI analyses. The standard fROI method requires human experts to meticulously examine and identify subject-specific fROIs within activation clusters. This process is time-consuming and heavily dependent on experts’ knowledge. Several algorithmic approaches have been proposed for identifying subject-specific fROIs; however, these approaches cannot easily incorporate prior knowledge of inter-subject variability. In the present study, we improved the multi-atlas labeling approach for defining subject-specific fROIs. In particular, we used a classifier-based atlas-encoding scheme and an atlas selection procedure to account for the large spatial variability across subjects. Using a functional atlas database for face recognition, we showed that with these two features, our approach efficiently circumvented inter-subject anatomical and functional variability and thus improved labeling accuracy. Moreover, in comparison with a single-atlas approach, our multi-atlas labeling approach showed better performance in identifying subject-specific fROIs.

## Introduction

The functional region of interest (fROI) approach has increasingly become an important methodology in functional magnetic resonance imaging (fMRI) studies [1–6]. In this approach, the fROIs are typically defined functionally in individuals. Specifically, a short functional localizer is typically acquired in each subject to define subject-specific fROIs according to their response profiles, and a main task is then used to test specific functional hypotheses concerning these regions [5,7,8]. The approach thereby circumvents inter-subject anatomical and functional (i.e., structural-functional correspondence) variability, and provides significant increases in the sensitivity and functional resolution of fMRI data analyses [4,9,10]. To exploit the advantages of the fROI approach, it is necessary to identify subject-specific fROIs. Typically, subject-specific fROIs are identified visually by human experts by examining activation clusters. Although this approach maximizes the advantage of human visual intelligence to discern inter-subject variability and identify fROIs, it is time-consuming and heavily depends on the experts’ knowledge, which is acquired only after extensive practice. Therefore, automated methods for identifying fROIs in individual activation images are highly desirable, particularly as the volume of data (i.e., sample size) in fMRI studies is increasingly large. In the present study, we aimed to develop an automatic approach to define fROIs based on the multi-atlas labeling (MAL) framework.

The development of automated algorithms to identify subject-specific fROIs is a difficult challenge because of the large inter-individual variability in anatomy and the lack of a clear mapping between function and structure. Several algorithms have been developed to identify fROIs in a semi-automated or fully automated manner. Generally, these methods can be classified into two categories. The first class of methods identifies activation foci as fROIs in individual brains by modeling the spatial variability of activation foci [11–14]. This type of approach could efficiently detect the foci of functional regions, but it does not provide other important information (e.g., shape and spatial extent). To obtain such information, another type of approach aims to detect the boundary of fROIs using conventional image segmentation methods. A diverse set of segmentation algorithms, such as region growing [15,16], watershed [17], ICA [18], and random walk [19], have been used to divide the activation image into distinct functionally coherent regions. However, for this class of methods, the decision as to which segmented regions should be labeled as a specific fROI still needs to be made by human experts.

Clearly, a better solution for automatic identification of fROIs is to incorporate the manually labeled atlases into the segmentation directly and learn the labeling criteria from these atlases. In this regard, the atlas-based segmentation method has performed remarkably well—without human intervention—in labeling anatomical brain regions (for a review, see [20]). An atlas-based approach has been developed for identifying subject-specific fROIs that utilizes a single atlas created from average of multiple subjects as a spatial constraint to guide the selection of the voxels belonging to each fROI. This approach is thereby named the Group-constrained Subject-Specific (GSS) method [1,21], and has been shown to be effective in identifying fROIs that have good spatial consistency across subjects. However, the single-atlas labeling (SAL) approach cannot accommodate the large inter-individual variability of fROIs and thus may lead to inaccurate delineation. The MAL approach has proved to be more robust and accurate than the SAL in segmenting anatomical brain regions, regardless of whether the atlas was created from an individual or a group of subjects (i.e., a group average atlas) [22–25]. In the MAL approach, an atlas consists of an intensity image and its corresponding manual segmentation. Given a target image, an initial segmentation can be propagated from each available atlas based on spatial correspondence, and then the multiple segmentations from a series of atlases can be fused together to produce the final segmentation. Although none of the single segmentations would be perfectly accurate, as long as these segmentations are not systematically biased, they can be combined to form a final segmentation that is more accurate than the segmentation from each individual [26–28]. Therefore, we expected that the MAL approach would provide better performance than the SAL approach in identifying subject-specific fROIs.

In a typical application of the MAL approach for labeling anatomical brain regions, the atlas images are generally registered to the target image and then the labels from each atlas are propagated to the target on the assumption of a one-to-one correspondence (OOC) at each point [22,24,29]. However, this is not the case for the fROI definition. Because of the large spatial variability in fROI location and extent across subjects, the functional images cannot be aligned well [30–33]. Thus it cannot be guaranteed that atlases and target images maintain good spatial correspondence after registration. Here, we searched for a suitable strategy for applying the MAL to label fROIs. Specifically, we tailored the MAL approach in two ways to boost the labeling accuracy of subject-specific fROIs.

First, we encoded each image atlas by randomized forest (RF) that was recently introduced to the MAL framework [34]. However, in contrast to Zikic et al.’s approach [34] that evaluates the performance of RF-based atlas-encoding scheme in labeling anatomical regions, here we applied it to label fROIs, which is a long-standing challenge because of large inter-individual variability of the fROIs. We demonstrated that the RF-based atlas-encoding scheme was better at accounting for large inter-individual variability of fROIs than image-based encoding scheme, and thus significantly leverage the accuracy of MAL in labeling fROIs. Second, an atlas selection procedure was employed to select an adequate set of atlases to facilitate the segmentation for each target image. As the fROI is highly variable across subjects, certain subjects in the database may be more appropriate as reference candidates than others for identifying the fROIs in a new subject [35–38]. Specifically, a widely used pattern-based similarity criterion was used to measure the similarity of two activation images and to rank the atlases for selection [35,39,40].

Recently, using 202 subjects, we created a functional atlas database for face recognition through manual delineation, comprising six widely studied face-selective regions (FSR) including the occipital face area (OFA), posterior and anterior fusiform face area (pFFA and aFFA), posterior continuation of the superior temporal sulcus (pcSTS), and posterior and anterior STS (pSTS and aSTS) [30]. In this study, we have quantified the inter-individual variability of each FSR and revealed that every FSR is highly variable in location and extent across subjects. Here, we used this functional atlas database to evaluate the proposed MAL approach. We first conducted a series of experiments to investigate the impact of the RF-based atlas-encoding scheme by comparing the RF encoding with the standard image based encoding. Then, we investigated the impact of atlas selection on the labeling accuracy of the fROI. Finally, we demonstrated that our MAL approach could provide better performance than the SAL approach (i.e., GSS) in identifying subject-specific fROIs.

## Methods

### Atlas database

The functional atlas database for face recognition, which is part of the Brain Activity Atlas project (http://www.brainactivityatlas.org), was used in this study. In the database, the subject-specific FSRs had been manually labeled for 202 participants (age: 18–23, 124 female) [30]. Specifically, a dynamic face localizer was used to localize the FSRs. The localizer used a blocked-design and consisted of four stimuli categories including faces, objects, scenes, and scrambled objects (for more details on the stimuli, see [41]). Each participant completed three runs in total, each of which lasted 3 min and 18 sec and contained two blocks for each condition. The MRI protocol was approved by the Institutional Review Board of Beijing Normal University. Written informed consent was obtained from all participants prior to the experiment in [30].

The functional images were analyzed using FEAT from FMRIB's Software Library (FSL, www.fmrib.ox.ac.uk/fsl). The statistical image was aligned to the individual’s structural images using FLIRT with 6 degrees of freedom, and then warped to the MNI152 template using FNIRT running with the default parameters. The FSRs were manually labeled for each subject based on the activation image from the contrast of faces versus objects. Specifically, the individual activation image was first thresholded at Z > 2.3 (p < 0.01, uncorrected), and then the OFA, pFFA, aFFA, pcSTS, pSTS, and aSTS were delineated by seven experts from suprathreshold voxels following a standard procedure. For more details on the atlas of the FSRs, see [30].

### MAL approach

#### Atlas encoding

In traditional MAL approaches, each atlas is encoded directly as a pair of image volumes (i.e., an intensity image and a label image). Then, the spatial correspondence between an atlas and target image is achieved through image registration. Because of the large inter-individual variability of the fROIs, the assumption of small and smooth deformation between each atlas and the target image, as required by image registration, is violated. As a result, the activation images from different subjects cannot be aligned well, which leads to mislabeling through label propagation based on the OOC assumption. An alternative method for coping with large inter-individual variability in atlas encoding is to learn a set of rules on the delineated fROI and then use these rules to label the activated clusters. To achieve this, we trained a RF classifier (implemented in scikit-learn, http://scikit-learn.org) to encode each atlas, and then used it to predict the labels for new subjects. The RF classifier, but not other classifiers, was employed because it could effectively handle the nonlinear relationship between the location and labels of voxels. Specifically, an RF was trained on one individual atlas (i.e., a pair of activation image and the corresponding label map). As the fROIs were delineated based mainly on the spatial locations of suprathreshold activation clusters, in the study we only used the spatial coordinates to encode the atlas. Thus, each activated voxel (Z > 2.3, p < 0.01, uncorrected) was treated as a sample, and its spatial coordinate was treated as feature. Each tree in the RF was trained to split the voxels at each node by maximizing the information gain in a randomly selected dimension of the coordinate. After training, a probabilistic association between the spatial coordinates and fROI labels was learned from each atlas by a RF. In particular, by combining trees constructed in randomly selected samples, the RF learned rules on fROI location and extent that were less affected by variation of the fROIs’ spatial properties (i.e., location, extent, and shape) across subjects compared with the typical image-based encoding method. For a new subject, a predicted label map could be generated by putting the activated voxels into each trained RF. Then, by averaging the predicted label maps from multiple RFs, a summarized probabilistic estimate was computed for each fROI. A majority voting rule was then used to determine the final label of each voxel.

#### Atlas selection

In addition to encoding of atlases with RF classifiers, an atlas selection procedure was also employed to account for the fROI variability. Because it would have been impractical to identify the optimal subset of atlases for each unlabeled subject by exhaustively searching all possible subsets in a large atlas database, we instead used a heuristic approach to select atlases according to the spatial similarity of activation pattern between unlabeled images and each atlas. Specifically, as the fROIs were defined from a specific functional contrast (e.g., the FSR was defined based on the contrast of faces > objects), the atlas similarity was measured based on the positive activation pattern alone. In detail, the Z values of negative activated voxels were assigned a value of zero and the similarity between a pair of subjects was calculated using Pearson’s correlation. The value “1” indicated that two subjects had the same activation pattern, and “-1” indicated that two subjects exhibited an opposite activation pattern. These similarity values were then used to rank the atlases. The top-ranked atlases were selected and used to generate a MAL estimate.

### MAL evaluation

We first assessed the effectiveness of the atlas-encoding scheme and the impact of atlas selection on the right OFA and pFFA, as these two FSRs were identified in most subjects with good consistency across experts [30]. A collective mask was created by merging rOFA and rpFFA from all atlases to provide a spatial constraint for the candidate voxels. Thus, only the pairs of activated voxels and corresponding labels (rOFA, rpFFA, or background) from the mask were used to train RFs. Second, we evaluated our approach for labeling several FSRs using the parameters optimized in the labeling of rOFA and rpFFA to test the stability of the MAL approach. Third, to assess the performance of our MAL approach, the achieved labeling accuracy was further compared with that from the SAL approach. Note that each RF consisted of 30 trees (T = 30), which were trained down to depth of 20 (D = 20) in the above analyses, as previous studies showed that this setting could achieve adequate performance [42–44]. Finally, we investigated the impact of the forest parameters on the performance of our MAL approach.

#### Effect of RF-based atlas encoding

The effect of the RF encoding scheme was assessed in two ways. First, if the RF-based scheme could better tolerate inter-individual variability, the coordinate-label relationships learned from a pair of atlases were expected to show higher similarity than the original labels. We therefore computed the similarity of each pair of atlases before and after RF encoding and compared them directly. Specifically, Dice’s coefficient was used to measure the similarity of two labels by calculating the spatial agreement between two labels relative to their total volume [45]. Second, a leave-one-subject-out cross-validation (LOSOCV) procedure was performed to estimate the labeling accuracy in which a single subject was iteratively left out as testing data, and the remaining subjects were used as the reference atlases. The accuracy of the MAL approach using the RF encoding scheme was measured with Dice’s coefficient, which calculates the spatial agreement between the automated labeling and the gold standard (i.e., manual labeling). For comparison, the typical image-based encoding scheme was also evaluated. Specifically, the labels from each atlas were directly transferred to the unlabeled subjects, assuming the spatial correspondence of the voxels in the stereotaxic space (i.e., MNI space). Then, the transferred labels were summarized by averaging the labels from all atlases, and a majority voting rule was used to determine the final label of each voxel. A paired t-test was used to assess the significance of the performance differences between the two atlas-encoding schemes.

#### Effect of atlas selection

To assess the impact of the atlas selection scheme, two experiments were conducted on the rOFA and rpFFA. First, the feasibility of the atlas selection scheme was validated. Given two atlases, it was expected that if the scheme was feasible, the RF trained on one atlas, which showed a more similar activation pattern to that of an unlabeled subject, would generate a more accurate labeling for the target. We therefore tested whether the degree of activation pattern similarity (i.e., rank) between an individual atlas and the target image could effectively predict its performance in labeling the fROIs in the target image. The similarity of activation patterns was computed within the mask generated in the atlas encoding section. Second, the optimal number of atlases to label the FSRs was determined by selecting an increasing number of the top-ranked atlases and then measuring the labeling accuracy. Here, we considered the possible range of values for the number of atlases: 1, 5, 10, 20, 30 … 200. To provide a baseline for the effect of atlas selection, for each number of atlases selected from the ranked list, 10 random sets of the same number of atlases were also selected to estimate the labels.

#### Comparison to the SAL approach

After assessing the impact of the RF-based atlas-encoding scheme and atlas selection procedure in accounting for the large variability of fROIs, we finally evaluated the performance of the MAL approach with both customizations in identifying fROIs compared with the SAL approach. Here, the number of selected atlases was set to 40, as this was the minimum number of atlases that yielded the best on-average performance in labeling rOFA and rpFFA. Meanwhile, because the number of selected atlases was optimized in the task of labeling the rOFA and rpFFA, we also conducted experiments on labeling several other regions, including the lOFA and lpFFA, and the FSRs from STS (i.e., pcSTS, pSTS, and aSTS) in both hemispheres to prevent bias and further validate the effectiveness of our scheme. As in labeling the rOFA and rpFFA, a collective mask was created for each experiment. We evaluated the labeling accuracy in two ways. First, we measured the overlap between automated labeling fROIs and the ground truth with Dice’s coefficient. Second, as the peak of an fROI is typically considered representative of the region, we measured the consistency of the peak location of the automatically labeled regions to the ground truth. The consistency of the peak location for each fROI was defined as the proportion of matched pairs across subjects. The same LOSOCV method was used to measure the labeling performance.

For comparison, the performance of an SAL approach (i.e., GSS) in labeling the same data was evaluated for each fROI [21]. In particular, fROIs were identified in three steps with the GSS approach. First, for each FSR, a probabilistic map was created by averaging the FSRs delineated in individual brains (Z > 2.3, uncorrected) to characterize the likelihood that a given voxel belonged to that FSR. Second, a maximum probability map (MPM) of FSRs was constructed to summarize the probabilistic maps of all FSRs into one volume. Specifically, the MPM was generated by setting the probability threshold of each FSR and then assigning each voxel to the FSR that had the highest probability. Finally, the labels from the MPM were used as group-level fROIs and were intersected with each individual’s activation map to generate subject-specific ROIs. The LOSOCV procedure was used to evaluate the performance of GSS. As the threshold used to make MPM had an impact on the extent of group-level fROI and in turn affected the labeling accuracy, the performance of the GSS was assessed with the average accuracy using different thresholds (i.e., 0, 0.1, and 0.2). The atlas selection procedure described above was also tested in GSS. The number of selected atlases was also set to 40 for comparison with the MAL.

Finally, as the goal of the fROI analysis was to study functional profiles of functional regions, we demonstrated that our MAL approach is advantageous in characterizing the activation of functional regions. The concordance of activation intensities from the automated fROIs and the manually labeled fROIs was evaluated. Specifically, for each subject, the mean activation intensities of rpFFA were extracted for the: (1) manually identified fROIs, (2) MAL-identified fROIs, and (3) fROIs identified by GSS-combined-with-atlas-selection (GSS+AS). Then, the activation intensities from MAL-identified and GSS+AS-identified fROIs were compared with those from the manually delineated fROIs through Pearson’s correlation.

#### Impact of forest parameters

In the above analysis, we assigned the parameters as T = 30 and D = 20. To assess the sensitivity of the model to the variation of the parameters, we conducted the same LOSOCV analysis as described above for defining FSRs using different parameter pairs, but varying T∈[10, 40] and D∈[10, 40] with a step of 5, and the results were evaluated using Dice’s coefficient. In these experiments, the number of selected atlases was assigned as 40.

## Results

### Atlas encoding

**Fig 1:**
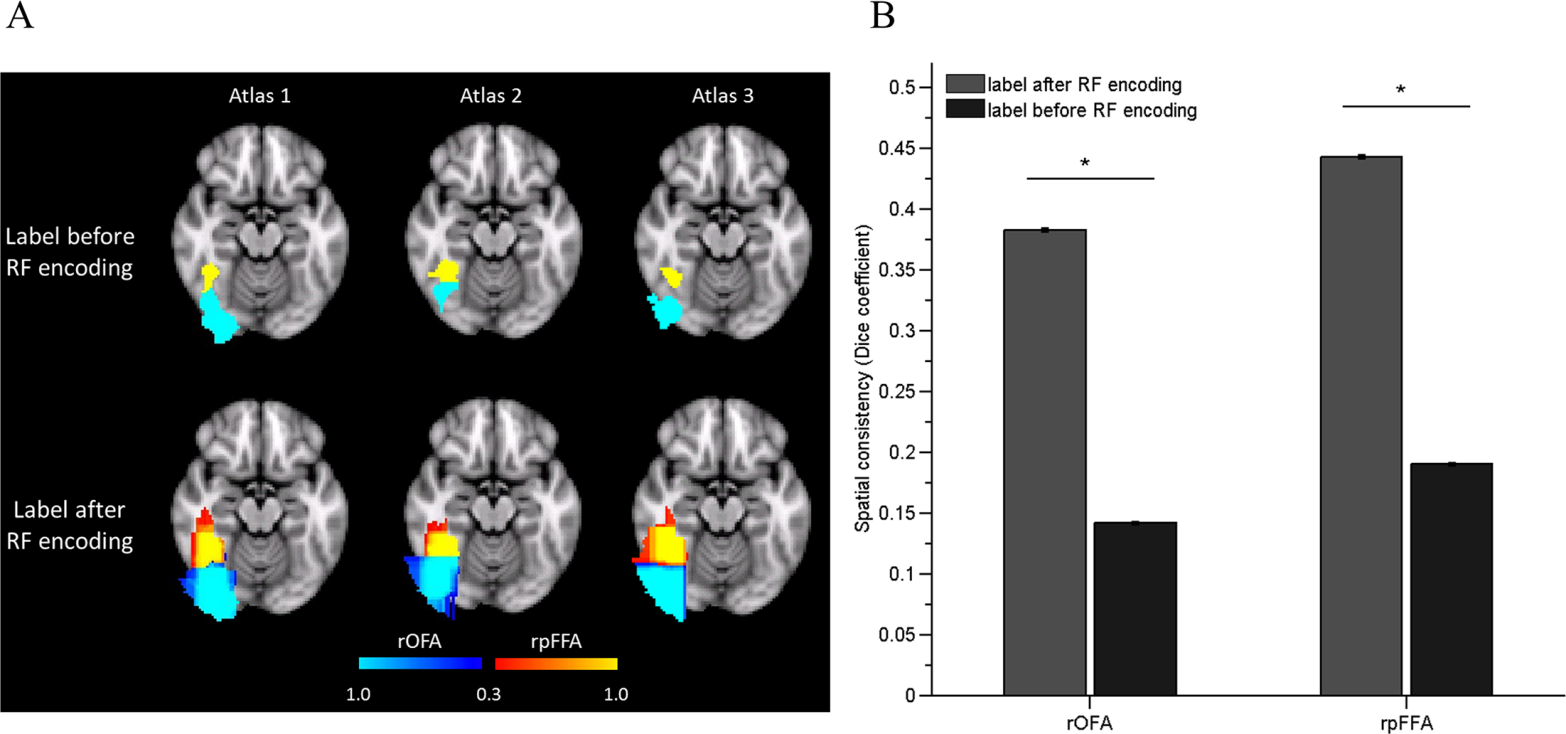
The RF encoding scheme alleviated the spatial correspondence between voxel and label pairs. A) Three samples of original labels and the corresponding RF encoded labels. The color bars indicate the probability of one voxel belonging to specific labels. The labels were overlaid on the MNI standard brain (z = -16). B) Inter-individual similarity of the label maps, before and after encoding by RF, measured by the Dice’s coefficient. The RF-encoded labels showed higher spatial consistency than the original labels across subjects.

The subject-specific FSRs were highly variable in their locations and extents (see S1 Fig). We encoded each atlas with an RF classifier to account for the large fROI variability across individuals. As shown in Fig 1A, an RF learned a probabilistic association between the coordinates and the labels. Particularly, by combining multiple trees constructed in a randomly partitioned feature space, the RF also provided an inference as to what extent an unlabeled voxel belonged to a label.

**Fig 2:**
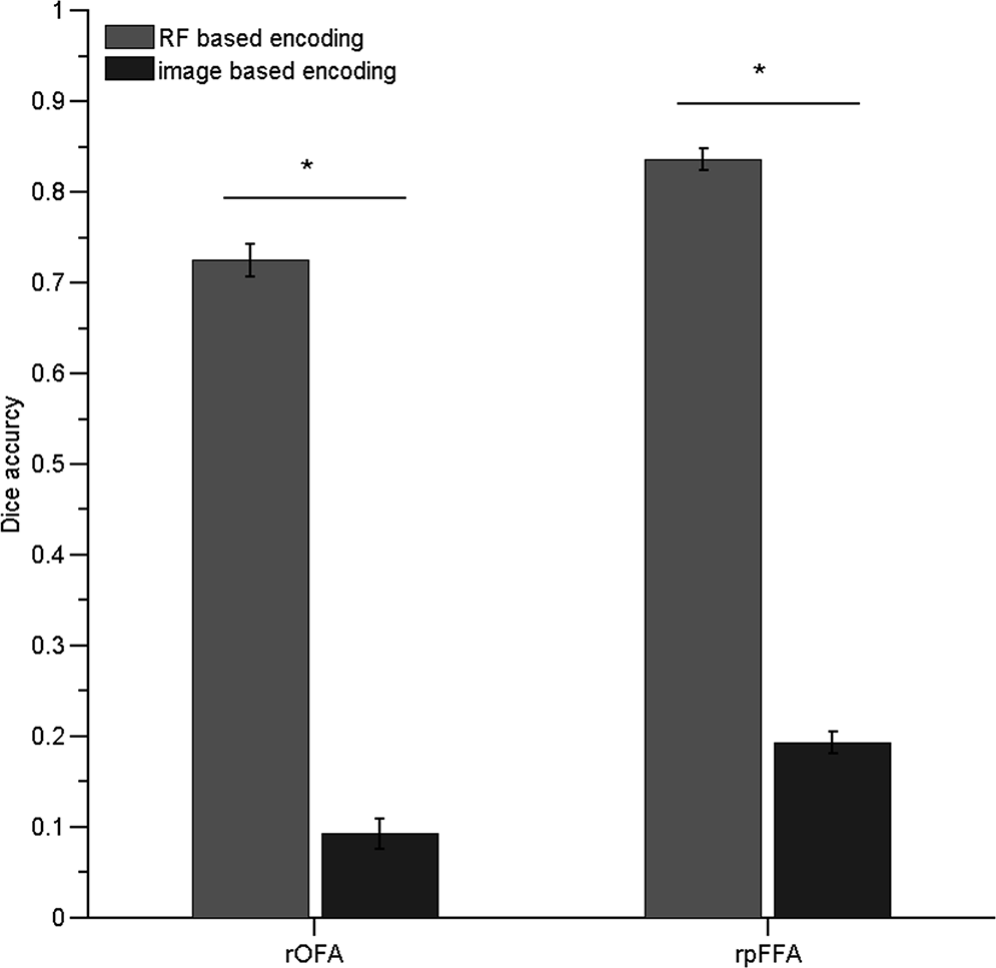
Effect of atlas encoding scheme on labeling accuracy measured by Dice’s coefficient. The RF encoding scheme showed better accuracy than the image-based encoding scheme in labeling the rOFA and rpFFA (paired t-test p < 0.00001).

Quantitative analysis revealed that, compared with the original labels, the RF-encoded labels showed improved consistency across atlases (Fig 1B, paired t-test, rOFA: p < 0.0001, rpFFA: p < 0.0001), indicating that the learned labels afforded a better tolerance to the spatial variability of the fROI, compared with the image-based atlas-encoding scheme. Furthermore, as shown in Fig 2, the labeling accuracy from the RF-based MAL was significantly higher than that from the image-based MAL (rpFFA, t(201) = 37.95, p < 0.0001; rOFA, t(201) = 20.03, p < 0.0001). Together, the results indicated that the RF is a suitable scheme for encoding atlases in an MAL approach for identifying fROIs.

### Atlas selection

**Fig 3:**
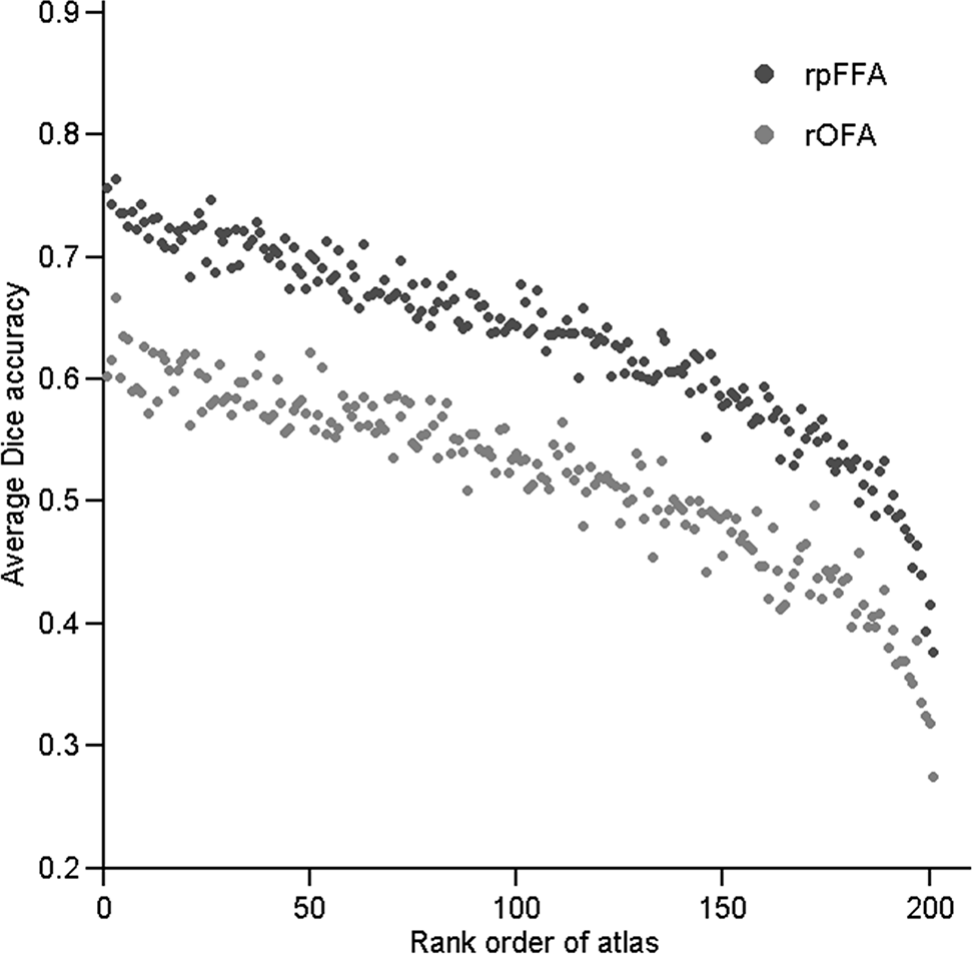
Relationship between average Dice accuracy obtained by individual atlases and their corresponding ranks. The rank for an atlas was assigned by computing the activation pattern similarity between the atlas and the target image. Dice’s index was iteratively computed with each subject as the target image and then averaged across all atlases (N = 202).

In addition to encoding of atlases with RFs, an atlas selection procedure was used to accommodate the fROI variability. The atlases that showed greater similarity of the activation pattern to that of the target subject were selected with higher priority. The feasibility of the atlas selection procedure was first validated by evaluating whether the rank of an individual atlas, which was defined according to the degree of its similarity to the target image, could effectively predict its performance in labeling the rOFA and rpFFA in target images. As illustrated in Fig 3, we plotted the average Dice accuracy derived from individual atlases in predicting different target images against their ranks. Clearly, there was a strong negative correlation between atlas rank and labeling accuracy (rOFA, r = -0.93; rpFFA, r = -0.94), indicating that high-ranked atlases were likely to produce a higher labeling accuracy.

**Fig 4:**
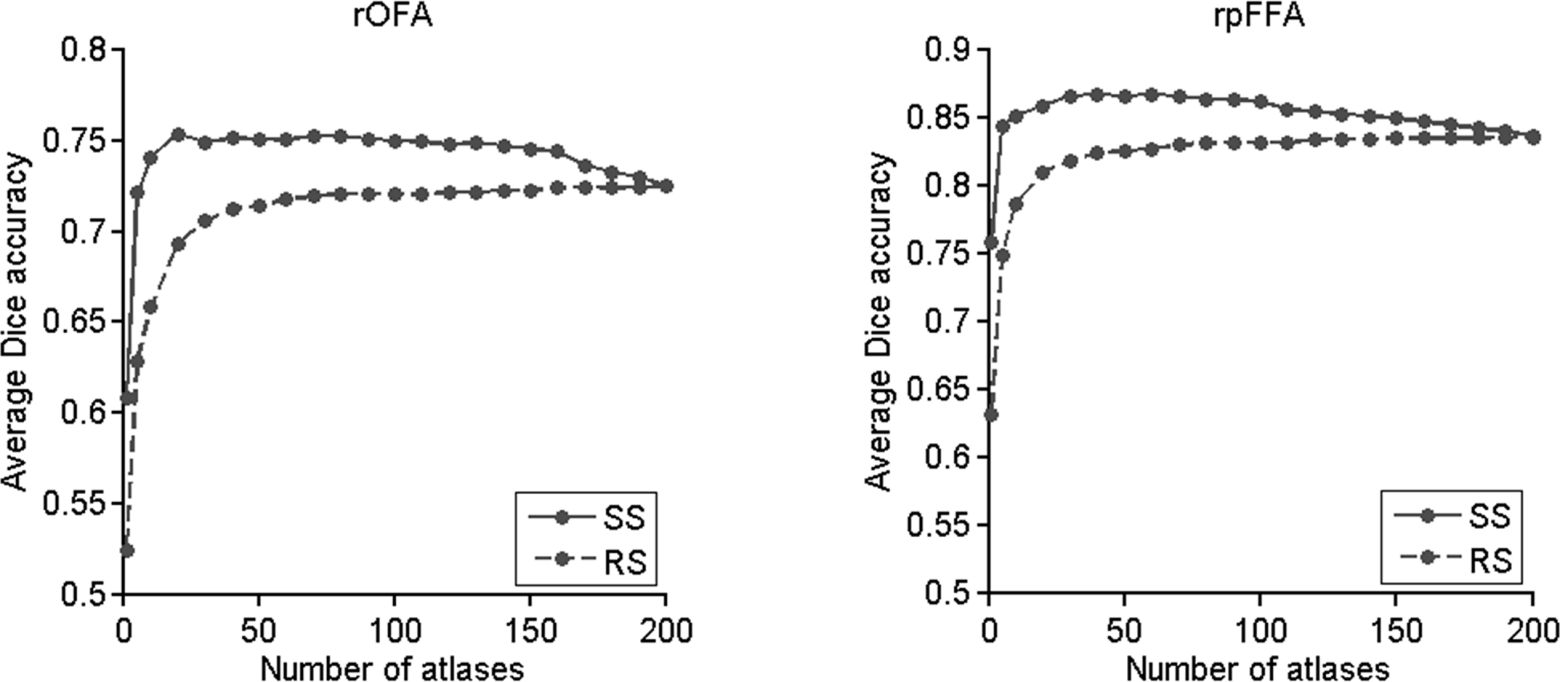
Effect of atlas selection on labeling accuracy measured by Dice’s coefficient. The labeling accuracy for the rOFA and rpFFA was computed with a different number of top-ranked and randomly selected atlases. SS, similarity based selection; RS, random selection.

We also investigated the effect of varying the number of selected atlases upon the final labeling. As the number of atlases increased, the labeling accuracy of the MAL constructed on the top-ranked atlases increased initially and then decreased gradually after reaching its maximum value at an optimal subset size (Fig 4). The optimal subset size varied for the two regions: approximately 20 for the rOFA and 40 for the rpFFA. The maximum average accuracy of two regions was obtained with a minimum subset of 40 atlases. A significant difference between the labeling accuracy from the optimal subset of atlases (i.e., 40 atlases) and that from the full database was observed for both fROIs (paired t-test, rOFA: p = 0.001; rpFFA: p < 0.0001). In contrast, the MAL constructed on the randomly selected atlases did not show such a pattern; its labeling accuracy increased continually until the whole database was used. Furthermore, the labeling accuracy from the randomly selected atlases was consistently lower than that from the top-ranked atlases in the subset size that ranged from 10 to 200. Similar results were observed when other similarity metrics (e.g., normalized mutual information) were applied to rank the atlases (see S2 Fig).

### Comparison to the SAL approach

**Fig 5:**
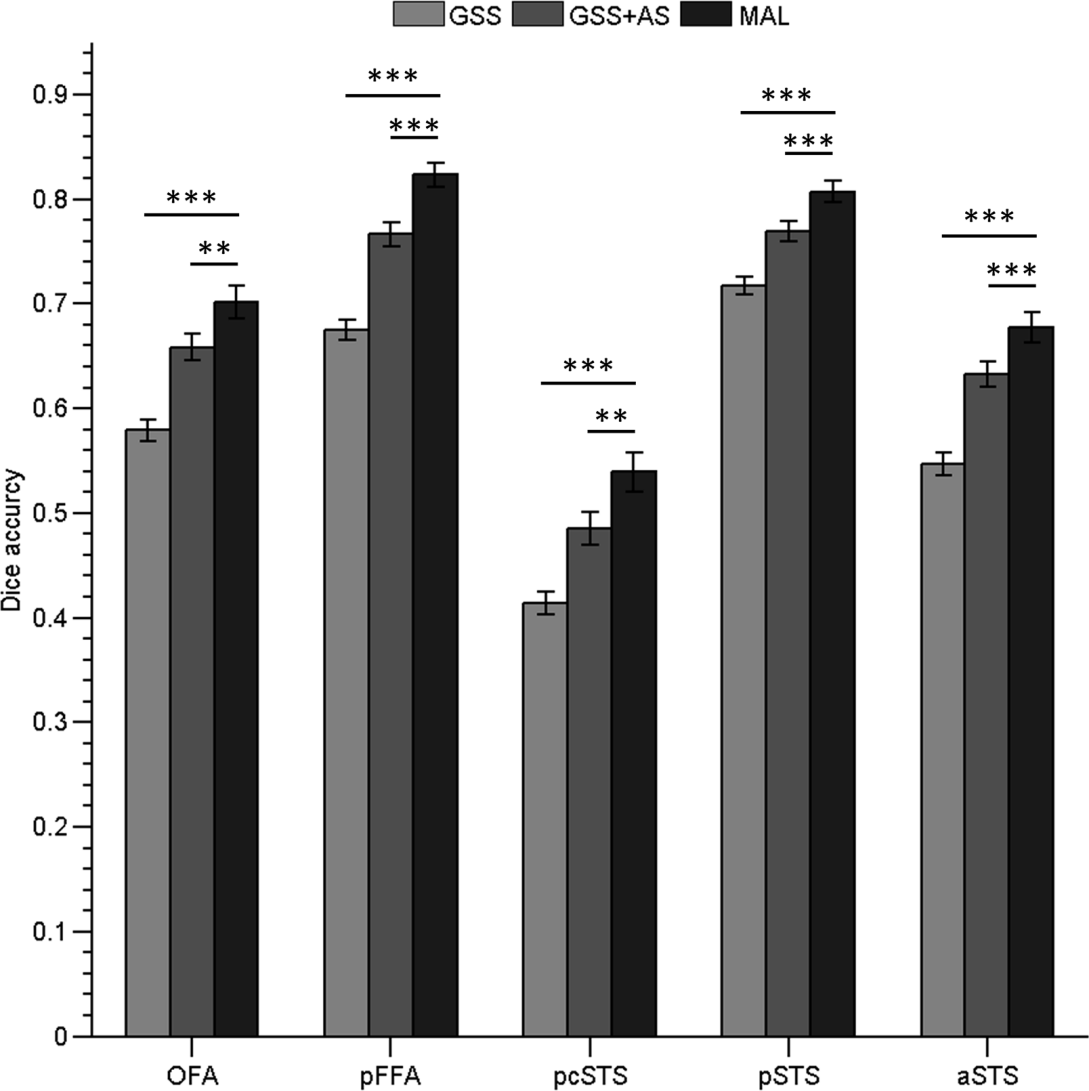
The performance of GSS, GSS+AS (GSS combined with atlas selection), and our MAL approach for identifying the OFA, pFFA, and other three independent FSRs located in the STS in both hemispheres. Each bar represents the Dice accuracy for an fROI averaged across hemispheres. *** indicates p < 0.0001, and ** indicates p < 0.001.

**Fig 6:**
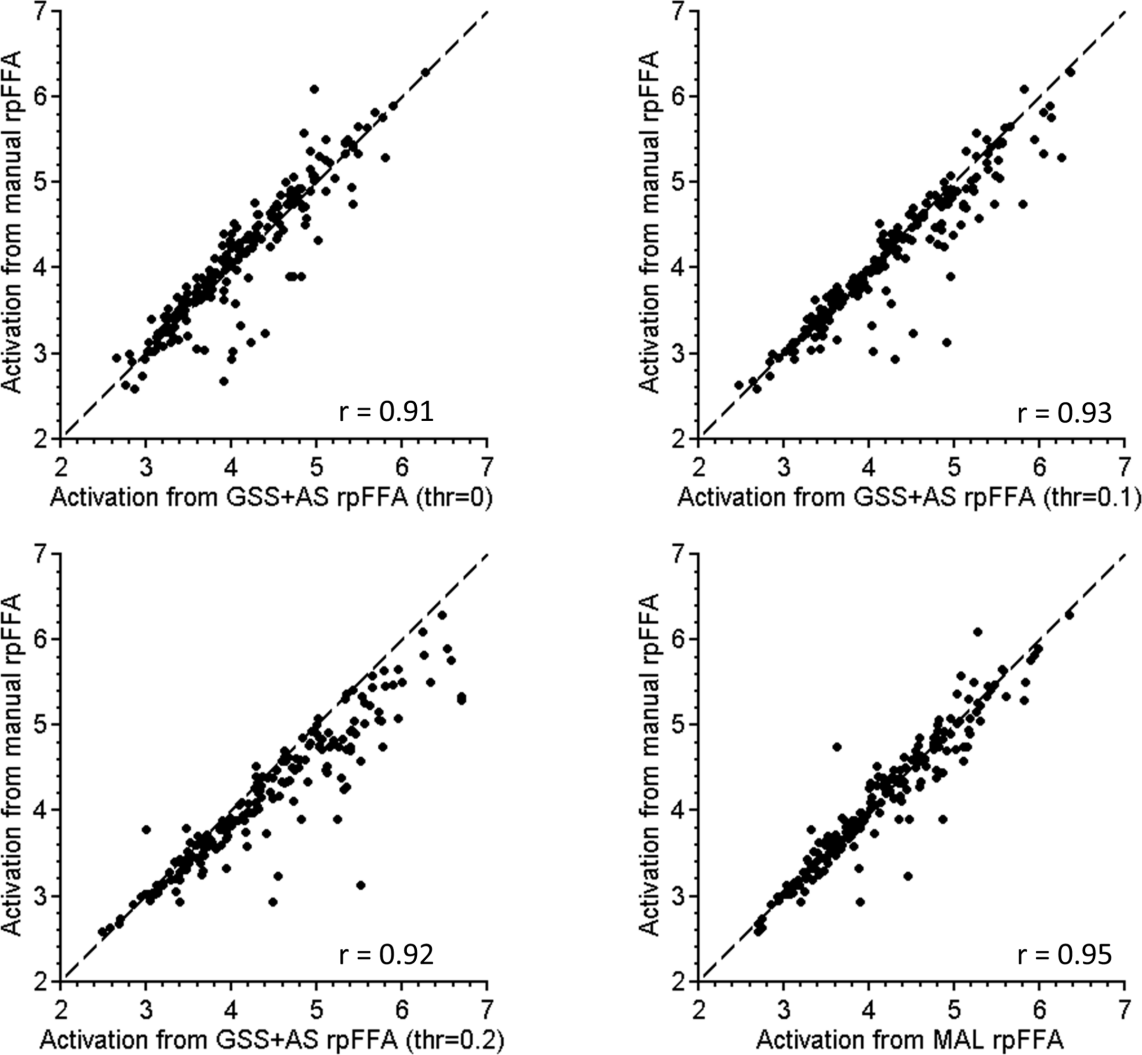
Comparison of the activation intensity (Z value for the contrast between faces and objects) of rpFFA identified by different automated approaches with that from manual delineation. Each point in the figure represents the activation intensity extracted from a subject.

After assessing the impact of the atlas encoding and selection scheme, we evaluated the performance of the MAL approach, paired with both features for labeling FSRs. As shown in Fig 5, our MAL approach exhibited an obvious advantage in labeling FSRs when compared with the GSS method. The MAL approach generally provided average accuracy gains of 12 percent and showed a 17-percent improvement in lpFFA (Table 1). Although the combination of the GSS approach with the AS approach (the GSS+AS approach) showed better labeling accuracy than the GSS alone, our MAL approach showed a further 5 percent increase in accuracy over the combination of GSS and AS (Fig 5 and Table 1). A paired t-test revealed that the MAL approach had significantly better labeling accuracy than the GSS+AS approach (rOFA: p < 0.0001, lOFA: p = 0.004, rpFFA: p < 0.0001, lpFFA: p < 0.0001, rpcSTS: p = 0.0003, lpcSTS: p = 0.014, rpSTS: p < 0.0001, lpSTS: p < 0.0001, raSTS: p = 0.0008, laSTS: p < 0.0001). In addition, the MAL approach showed significantly better performance in recovering the peak location than other methods (Table 2). The consistency of the peak location identified by the MAL approach was significantly higher than that from the GSS+AS approach for most FSRs (one-tailed paired t-test, rOFA: p = 0.03, lOFA: p < 0.0001, rpFFA: p = 0.004, lpFFA: p < 0.0001, rpcSTS: p = 0.08, lpcSTS: p = 0.36, rpSTS: p = 0.03, lpSTS: p = 0.04, raSTS: p = 0.3, laSTS: p < 0.0001). Moreover, in comparison with the fROIs from the GSS+AS method, the MAL-identified fROIs were more consistent with the manually delineated fROIs with regard to their activation intensities. As shown in Fig 6, a stronger correlation was observed between the activation intensities extracted from the manually delineated rpFFA and the MAL-identified rpFFA (p < 0.01 examined by Steiger’s Z test). In summary, these results indicate significantly better performance in identifying subject-specific fROIs for our multi-atlas labeling approach compared to the GSS+AS method.

**Table 1:** Labeling performance (mean Dice’s coefficient ± standard error) from different methods in each hemisphere.

ROI	L/R	GSS	GSS + AS	MAL
OFA	R	0.64±0.01	0.71±0.02	**0.75±0.02**
	L	0.52±0.01	0.60±0.02	**0.67±0.02**
pFFA	R	0.74±0.01	0.82±0.01	**0.87±0.01**
	L	0.61±0.01	0.71±0.02	**0.78±0.02**
pcSTS	R	0.45±0.01	0.54±0.02	**0.6±0.02**
	L	0.37±0.01	0.43±0.02	**0.48±0.03**
pSTS	R	0.77±0.01	0.80±0.01	**0.83±0.01**
	L	0.67±0.01	0.73±0.01	**0.78±0.02**
aSTS	R	0.59±0.01	0.67±0.01	**0.69±0.02**
	L	0.51±0.01	0.60±0.02	**0.66±0.02**

OFA, occipital face area; pFFA, posterior fusiform face area; pcSTS posterior continuation of the superior temporal sulcus (STS); pSTS, posterior STS; aSTS, anterior STS; L, left hemisphere; R, right hemisphere. The number of selected atlases in MAL and GSS+AS approaches was set to 40. Numbers in bold indicate that MAL showed significantly greater accuracy than the GSS + AS method, one-tailed paired t-test, p < 0.05.

**Table 2:** Accuracy of peak detection with different methods (mean accuracy± standard error).

ROI	L/R	GSS	GSS + AS	MAL
OFA	R	0.60±0.03	0.67±0.03	**0.70±0.03**
	L	0.38±0.02	0.42±0.02	**0.66±0.03**
pFFA	R	0.81±0.02	0.84±0.02	**0.89±0.02**
	L	0.58±0.03	0.66±0.03	**0.83±0.03**
pcSTS	R	0.43±0.03	0.50±0.03	0.53±0.04
	L	0.41±0.02	0.47±0.03	0.48±0.04
pSTS	R	0.82±0.02	0.84±0.02	**0.87±0.02**
	L	0.69±0.03	0.72±0.03	**0.74±0.03**
aSTS	R	0.54±0.03	0.64±0.03	0.65±0.03
	L	0.45±0.02	0.55±0.03	**0.66±0.03**

The number of selected atlases in MAL and GSS+AS approaches was set to 40. Numbers in bold indicate that MAL showed significantly higher accuracy than the GSS + AS method, one-tailed paired t-test, p < 0.05.

### Impact of forest parameters

**Fig 7:**
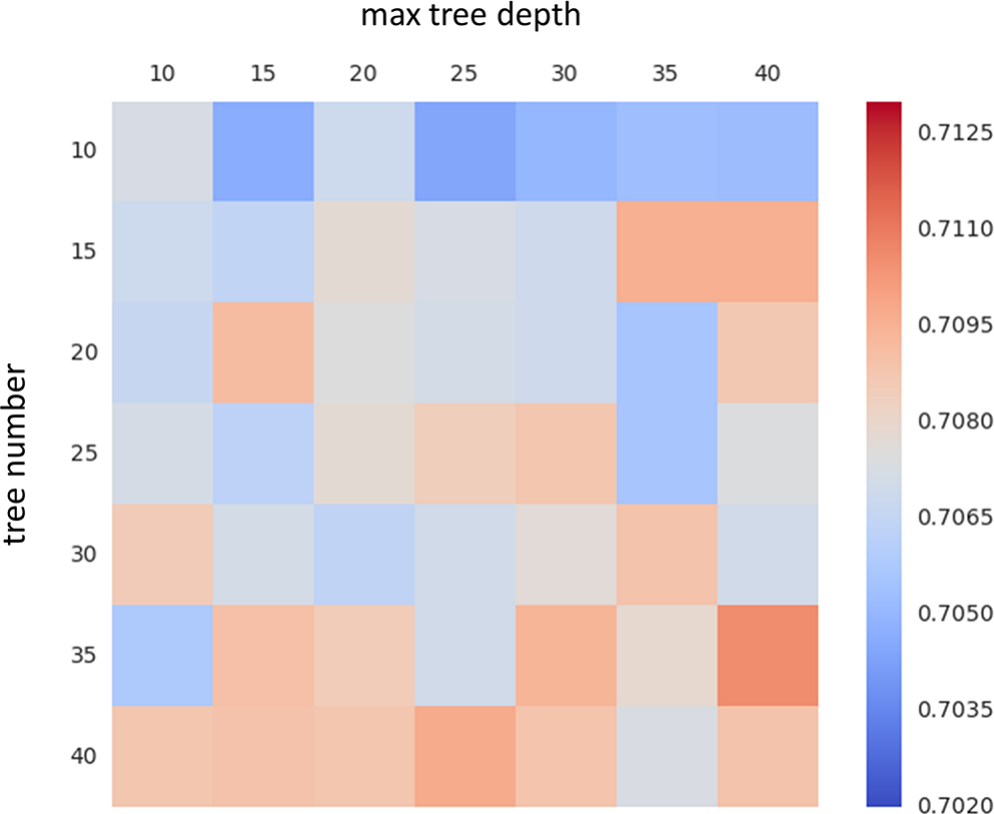
Influence of forest parameters on fROI labeling results. Each element in the matrix represents an averaged Dice accuracy across all fROIs from the RFs trained with the specific pair of maximum tree depth and tree number in the forest.

We assessed the sensitivity of labeling accuracy to variation of forest parameters by varying T∈[10, 40] and D∈[10, 40]. A LOSOCV was performed for each FSR for each combination, and the labeling accuracy was measured for each combination using Dice’s coefficient. As shown in Fig 7, the labeling accuracy was not sensitive to the variation of the forest parameters, indicating that the parameters used in the above analysis were appropriate.

## Discussion

In this study, we developed a multi-atlas labeling approach to identify subject-specific fROIs automatically. Compared with widely used single-atlas approaches, our MAL method achieved higher accuracy in identifying face-selective regions in individual subjects. In particular, to address the large variability of fROIs across subjects, an RF-based atlas-encoding scheme was used to represent the association between the spatial coordinates and fROI labels, and an activation pattern-based atlas selection procedure was used to select the atlases similar to the target. These two features significantly improved the labeling accuracy of the MAL.

Consistent with intuition that the MAL approach usually works better than the SAL approach [22–25,28], here we provide the first empirical evidence qualifying the advantage of the MAL approach over the SAL approach (i.e., the GSS method) in identifying subject-specific FSRs in the human brain. In addition, as previous studies have shown that the FSRs can be reliably identified with different localizer tasks [46], the learned model in this study can be directly adopted in other studies for identifying FSRs, regardless of the different paradigms and stimuli used in localizer. Furthermore, the approach can be in principle extended to identify fROIs other than FSRs with typical functional localizer data when manually delineated fROIs are available for training RFs. As the amount of fMRI data collected in scientific and clinical studies (e.g., Human Connectome Project) is dramatically increasing, automated and efficient tools to study brain functional regions are increasingly necessary. Therefore, our MAL approach provides a powerful tool to identify subject-specific fROIs in a large number of subjects, thus facilitating the probing of the structural, functional, and connectional properties of these regions with higher sensitivity.

Two important features were incorporated into the traditional MAL approach to identify subject-specific fROIs based on activation images. The first feature was related to the atlas encoding. In typical applications of the MAL approach for labeling anatomical regions, the labels for the target image are often transferred from the atlases in terms of the spatial correspondence, which is achieved through image registration [22,24,29]. Nevertheless, because of the large spatial variability of fROIs, it is not feasible to align pairs of activation images to have adequate spatial correspondence even when using the state-of-the-art curvature-based registration approach of cortical folding [47]. To overcome this difficulty, we trained a classifier (i.e., an RF) to encode each atlas. The RFs learned the mapping between voxel coordinates and fROI labels in a probabilistic manner, and thus alleviated the requirements for the OOC between the voxel/label pairs across subjects. Our classifier-based atlas-encoding scheme significantly improved the labeling accuracy compared to the image-based encoding scheme, suggesting that this scheme is suitable for representing the spatial distribution of functional regions.

The second feature that we implemented was related to the number of atlases to be used in labeling a new subject’s fROIs. The impact of atlas selection within the MAL framework has been widely acknowledged in the segmentation of anatomical regions. For example, a previous study [35] revealed that image-based atlas selection improves the accuracy of the segmentations of subcortical regions. Here, a similar atlas selection procedure was adopted. Consistent with previous findings on labeling anatomical regions [35,48], we observed that simply using the full database did not necessarily lead to the highest accuracy; rather, the labeling accuracy was improved through atlas selection. In contrast, the atlas selection procedure showed different effects for identification of fROIs compared to those for identification of anatomical regions. First, labeling of fROIs generally requires more atlases to achieve the best labeling compared to the labeling of anatomical regions. On average, 40 atlases are needed in identifying fROIs, whereas 8 to 15 atlases are required to label anatomical regions [35,48,49]. Second, the optimal set of atlases was shown to provide a greater advantage in accuracy (relative to the full database) when labeling fROIs (~ 4%) than when labeling anatomical regions (~ 2%) [48,49]. This difference may be attributed to the larger inter-individual variability of fROIs compared with that of anatomical regions.

Although our MAL approach showed better performance than the SAL approach in identifying subject-specific fROIs, several aspects can be improved for its future application. First, in addition to the location of each voxel, the local properties of each voxel other than activation (e.g., temporal response profiles) should be taken into account in the atlas encoding. As more properties are encoded, better performance of the MAL approach may be obtained. Second, although activation pattern-based atlas selection is reasonably simple and yielded satisfied results, other similarity metrics may provide better performance. For example, the hyperaligment method can be used to measure the similarity between target subjects and selected atlases [50,51]. Third, rather than using the majority voting rule for label fusion, more sophisticated fusion schemes can be used to improve the labeling accuracy. For example, the label can be fused using globally or locally weighted voting rules [52]. Finally, more advanced methods, such as patch-based MAL approaches [53,54], which are originally developed to label anatomical images, may be applied to label fROIs as well. Future work is needed to evaluate the applicability of these methods and compare them with ours.

The code used in this work has been made available for public use (see https://github.com/sealhuang/multi-atlas-froi).

## Supporting Information

S1 Fig Sample delineated RFs in the right hemisphere from 8 randomly selected brains, overlaid on the cortical surface. Because of space limitations, only the ventral view is presented. OFA and pFFA are shown in red and blue.
(TIF)

S2 Fig Effect of atlas selection on accuracy (as determined by Dice’s index). For each number of atlases, the labeling accuracy for the rOFA and rpFFA was computed using the top-ranked subset of atlases based on the normalized mutual information (NMI). The average Dice’s coefficient was estimated by a leave-one-subject-out cross-validation procedure for each subset of atlases.
(TIF)
