# Supplementary figures and images for: A Multi-Atlas Labeling Approach for Identifying Subject-Specific Functional Regions of Interest
Source: PLoS One. 2016 Jan 21;11(1):e0146868. doi: 10.1371/journal.pone.0146868 (PMC4721956; doi:10.1371/journal.pone.0146868)

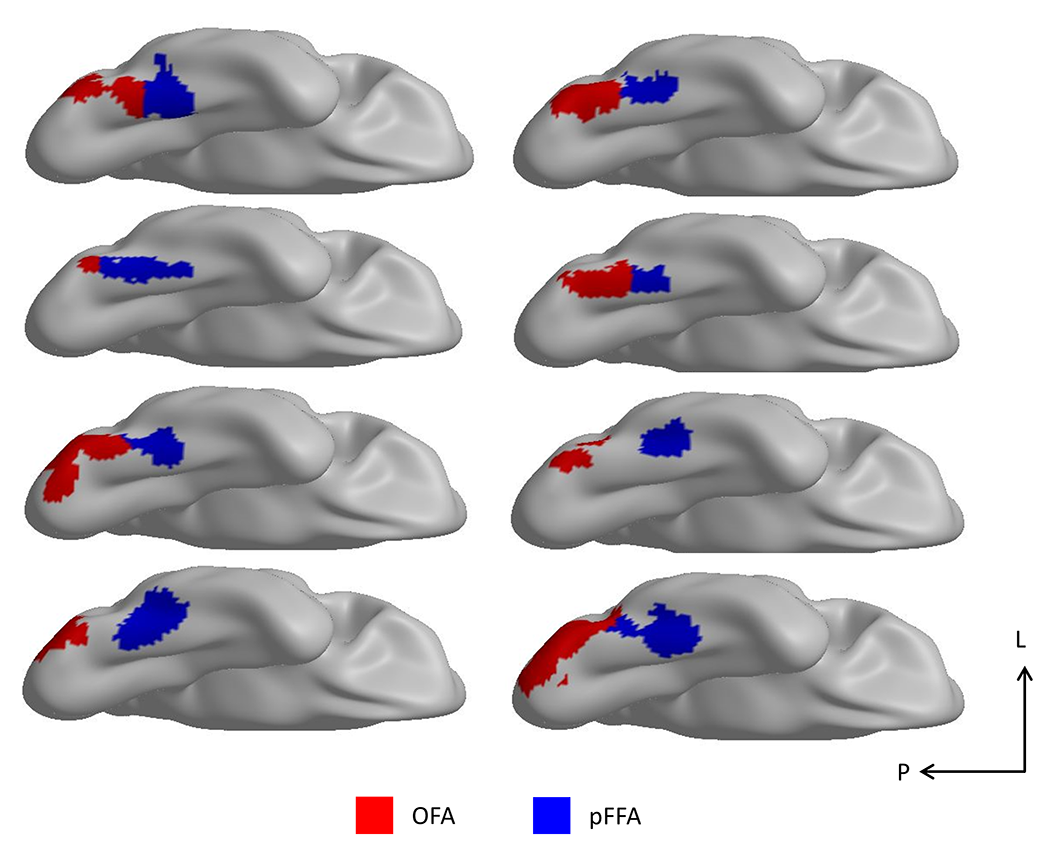

Supplement: S1 Fig — Because of space limitations, only the ventral view is presented. OFA and pFFA are shown in red and blue. (TIF) [file pone.0146868.s001.tif]

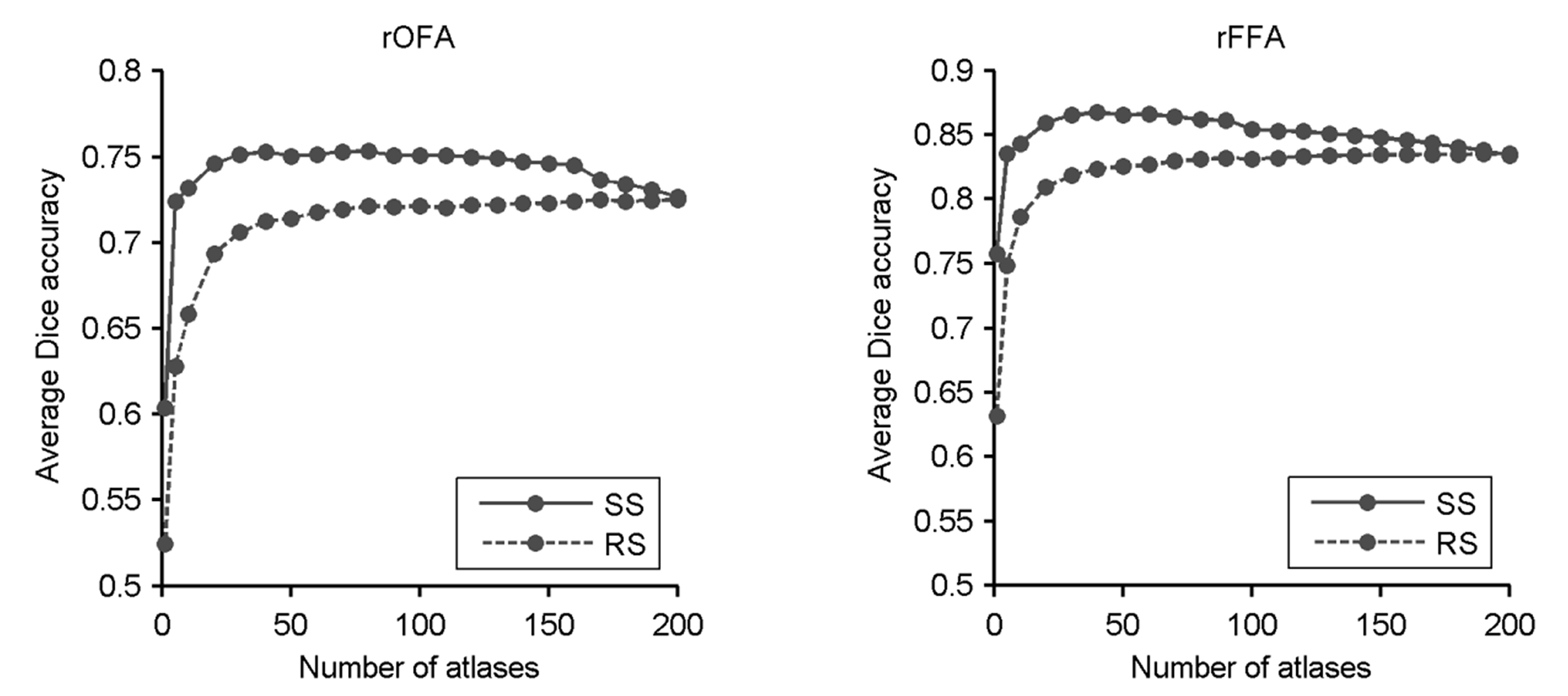

Supplement: S2 Fig — For each number of atlases, the labeling accuracy for the rOFA and rpFFA was computed using the top-ranked subset of atlases based on the normalized mutual information (NMI). The average Dice’s coefficient was estimated by a leave-one-subject-out cross-validation procedure for each subset of atlases. (TIF) [file pone.0146868.s002.tif]
